# Switchable Wettability and Adhesion of Micro/Nanostructured Elastomer Surface via Electric Field for Dynamic Liquid Droplet Manipulation

**DOI:** 10.1002/advs.202000772

**Published:** 2020-08-02

**Authors:** Yan Li, Jinrong Li, Liwu Liu, Yufeng Yan, Qiuya Zhang, Na Zhang, Linlin He, Yanju Liu, Xiaofang Zhang, Dongliang Tian, Jinsong Leng, Lei Jiang

**Affiliations:** ^1^ Key Laboratory of Bio‐Inspired Smart Interfacial Science and Technology of Ministry of Education School of Chemistry Beihang University Beijing 100191 P. R. China; ^2^ National Key Laboratory of Science and Technology on Advanced Composites in Special Environments Harbin Institute of Technology Harbin Heilongjiang 150080 P. R. China; ^3^ Department of Astronautical Science and Mechanics Harbin Institute of Technology Harbin Heilongjiang 150001 P. R. China; ^4^ School of Mathematics and Physics University of Science and Technology Beijing Beijing 100083 P. R. China; ^5^ Beijing Advanced Innovation Center for Biomedical Engineering Beihang University Beijing 100191 P. R. China; ^6^ Technical Institute of Physics and Chemistry Chinese Academy of Sciences Beijing 100191 P. R. China

**Keywords:** droplet manipulation, electric fields, micro/nanostructured surfaces, switchable wettability, tunable adhesion

## Abstract

Dynamic control of liquid wetting behavior on smart surfaces has attracted considerable concern owing to their important applications in directional motion, confined wetting and selective separation. Despite much progress in this regard, there still remains challenges in dynamic liquid droplet manipulation with fast response, no loss and anti‐contamination. Herein, a strategy to achieve dynamic droplet manipulation and transportation on the electric field adaptive superhydrophobic elastomer surface is demonstrated. The superhydrophobic elastomer surface is fabricated by combining the micro/nanostructured clusters of hydrophobic TiO_2_ nanoparticles with the elastomer film, on which the micro/nanostructure can be dynamically and reversibly tuned by electric field due to the electric field adaptive deformation of elastomer film. Accordingly, fast and reversible transition of wetting state between Cassie state and Wenzel state and tunable adhesion on the surface via electric field induced morphology transformation can be obtained. Moreover, the motion states of the surface droplets can be controlled dynamically and precisely, such as jumping and pinning, catching and releasing, and controllable liquid transfer without loss and contamination. Thus this work would open the avenue for dynamic liquid manipulation and transportation, and gear up the broad application prospects in liquid transfer, selective separation, anti‐fog, anti‐ice, microfluidics devices, etc.

## Graphical abstract

Dynamic liquid droplet manipulation and transportation are demonstrated on the superhydrophobic micro/nanostructured elastomer surface via electric field, based on electric adaptive switchable wettability and tunable adhesion. This work provides a new route for precise liquid manipulation and transfer, and is promising in designing and constructing smart interface materials.

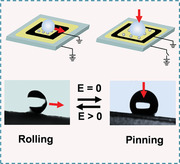

## 1 Introduction

Controllable liquid droplet manipulation and transportation have attracted more and more attention because of its advanced applications in liquid transfer,^[^ ^1^ ^]^ liquid printing,^[^ ^2^ ^]^ drug delivery,^[^ ^3^ ^]^ microfluidic devices,^[^ ^4^ ^]^ and so on. Up to now, a series of studies, including varying surface structure geometry, arranging asymmetric placement of surface structures, and changing chemical composition, etc.,^[^ ^5^ ^]^ had been done to achieve the imbalanced surface energy and Laplace pressures of surface for liquid droplet driving and controllable liquid droplet transportation with the cooperation of external stimuli,^[^ ^6^ ^],^ e.g., temperature,^[^ ^7^ ^]^ light,^[^ ^8^ ^]^ electricity,^[^ ^9^ ^]^ magnetism,^[^ ^10^ ^]^ mechanical stress,^[^ ^11^ ^]^ and so on. Among all these cases, mechanical stretch is an in situ, eco‐friendly, biocompatible method to control the wetting behavior of droplet dynamically by tuning micro/nanostructures of the surface,^[^ ^12^ ^]^ and the controllable motion state of droplet on the elastomer film in the presence of lubricant has been realized.^[^ ^13^ ^]^ Despite much progress in this field, the maneuverability of dynamic liquid droplet manipulation and transportation with rapid response, no loss, and anti‐contamination is still challenging.

Dielectric elastomer is a typical soft electroactive material that can fastly deform sustainably when subjected to external electric field stimuli due to the action of Maxwell stresses (the response time of dielectric elastomer is in the order of milliseconds, mostly < 1 ms).^[^ ^14^ ^]^ Due to the excellent properties of large actuation strains, high energy densities and high efficiencies, dielectric elastomer materials have been widely used to design and manufacture sensors and actuators, which have promising applications in soft robotics,^[^ ^15^ ^]^ and also provide the feasibility to transform the micro/nanostructures on the elastomer surface for dynamic liquid droplet manipulation and liquid transportation. However, there are still difficulties to solve the problems on loss and contamination of the transported liquid.

Herein, we demonstrate a strategy to dynamically manipulate liquid droplet transportation on the electric field responsive elastomer surface with adaptive micro/nanostructures, which is constructed by introducing hydrophobic clusters of TiO_2_ nanoparticles on the elastomer surface. The spacing of the hydrophobic micro/nano‐structured clusters of nanoparticles on the surface can be adjusted by voltage due to the electric field‐induced deformation of dielectric elastomer film, and thus the wetting state and adhesion of the surface can be controlled correspondingly. Accordingly, the droplet can be manipulated to implement some intricate dynamic behaviors, such as jumping and pinning, catching and releasing, and controllable liquid transfer, on the electrically controlled superhydrophobic surfaces, which can be utilized for uncontaminated and lossless liquid transportation. The electric field‐induced stretchable device with fast response, high elastic mechanical responses, easy to control, no manual operation and easy to combine with other devices would gear up broad application prospect in the fields of liquid transfer, selective separation, anti‐fog and anti‐ice, micro‐reactor, and micro‐fluid technology.

## 2 Results and Discussion

**Figure 1:**
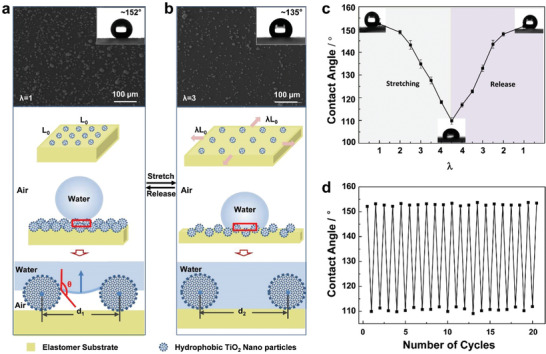
Controllable wettability of the micro/nanostructured elastomer film. SEM images and adaptive water wetting states of the micro/nanostructured elastomer film a) before and b) after equi‐biaxially stretch with stretch ratio *λ* = 3. Hydrophobic TiO_2_ nanoparticles are clustered on the elastomer surface and forming the micro/nanostructured elastomer film, showing superhydrophobic performance with water contact angle (CA) of ≈152°. When the elastomer film is stretched with *λ* = 3, water CA on the micro/nanostructured surface decreased to ≈135° accompanying with the increased distance between the micro/nanostructured clusters. c) Surface wettability to water can be reversibly tuned by stretching and releasing the elastomer film between *λ* = 1 and *λ* = 4.5. d) The switch of water CA between ≈110° (*λ* = 4.5) and more than 150° (*λ* = 1) is reversible and stable even after the repeating for 20 times. The results show that the wettability can be controlled reversibly through mechanical stretch of the micro/nanostructured elastomer film.

It has been revealed that surface wetting behavior is determined by the surface free energy and is further enhanced by micro/nanoscale topographical features.^[^ ^16^ ^]^ On a low free energy surface, the existent micro/nanostructures can provide an effective method of tuning surface wettability from hydrophobicity to superhydrophobicity. In this work, hydrophobic clusters of TiO_2_ nanoparticles with the diameter of ≈25 nm are firstly constructed on the elastomer surface to form the superhydrophobic micro/nanostructured elastomer film. The micro/nanostructured clusters on the surface are uniform with the diameter of 13.4 ± 1.5 µm and the distance of 13.5 ± 3.5 µm, and the micro/nanostructured elastomer film surface is superhydrophobic with water contact angle (CA) of ≈152° (**Figure** **1**a). When the elastomer film is equi‐biaxially stretched with a stretch ratio (*λ*) from *λ* = 1 to *λ* = 3, the distance of the micro/nanostructured clusters increased from 13.5 ± 3.5 to 24.0 ± 3.7 µm, accompanying with a decrease of the water CA on the micro/nanostructured surface from ≈152° to ≈135° (Figure 1b). In the stretching process, we found that solid–liquid contact fraction will increase with the stretch, which have important influence on the surface wettability. For partially wetting droplet suspended on micro/nanostructured superhydrophobic surface, according to the Cassie–Baxter equation.^[^ ^17^ ^]^

(1) $$\begin{array}{c} \cos\theta =r_{f}\;f_{s}\left(1+\cos\theta_{Y}\right)-1 \end{array}$$

where *r*_f_ is the roughness factor, *f*_s_ is the solid–liquid contact fraction defined by the ratio of projected wet area to the unit area, *θ*_Y_ is the Young's CA and *θ* is the apparent CA, the stretch obviously induces the increase of solid–liquid contact fraction *f*_s_ and results in a decrease of the apparent CA *θ*. The generation of the difference in water CA before and after stretching can even change wetting state from Cassie state to Wenzel state.

Further results indicate that the distance of the micro/nanostructured clusters can be adjusted from 13.5 ± 3.5 to 87.4 ± 9.1 µm after the equi‐biaxially stretch of the micro/nanostructured elastomer film increases from *λ* = 1 to *λ* = 9 (Figure S1, Supporting Information). Correspondingly, the water CA reduces from ≈152° to ≈89°, which is close to the water CA of the unmodified elastomer film, and will not decrease after further stretch.

For the micro/nanostructured elastomer film with the micro/nanostructured clusters radius (*r*) of 6.7 ± 1.5 µm and the distance of micro/nanostructured clusters (*d*) when the droplet just contacts with elastomer film, the critical distance of micro/nanostructured clusters, *d*_c_, can be described as

(2) $$\begin{array}{c} d_{c}=\frac{2\;\mathrm{rcos}\theta }{\sin\theta -1}\; \end{array}$$

By calculation, when the distance of micro/nanostructured cluster (AB) is 22.3 ± 5.0 µm, i.e., the center distance of micro/nanostructured cluster (O_1_O_2_) is 35.7 ± 5.0 µm, the droplet will contact and just wet the elastomer substrate film, indicating the wetting state changes from Cassie state to Wenzel state and the droplet adheres to the substrate (Figure S2, Supporting Information).

Based on the above experiment results and analysis, to achieve the reversible wettability switch between superhydrophobicity and hydrophobictiy on the micro/nanostructured elastomer surface, water droplet contact with the elastomer film is supposed to be avoided. In this work, the micro/nanostructured elastomer film shows good reversible wettability transition when equi‐biaxially stretch *λ* < 4.5, indicating that it is the suitable area for the goal (Figure 1c). The micro/nanostructured elastomer film is durable, and the CA switched between ≈110° and more than 150° is reversible and stable even after repeating the switch of stretch between *λ* = 1 and *λ* = 4.5 for 20 times (Figure 1d). Thus the controllable wettability switch between superhydrophobicity and hydrophobictiy is obtained on the micro/nanostructured elastomer film owing to the reversible micro/nanostructure transition.

**Figure 2:**
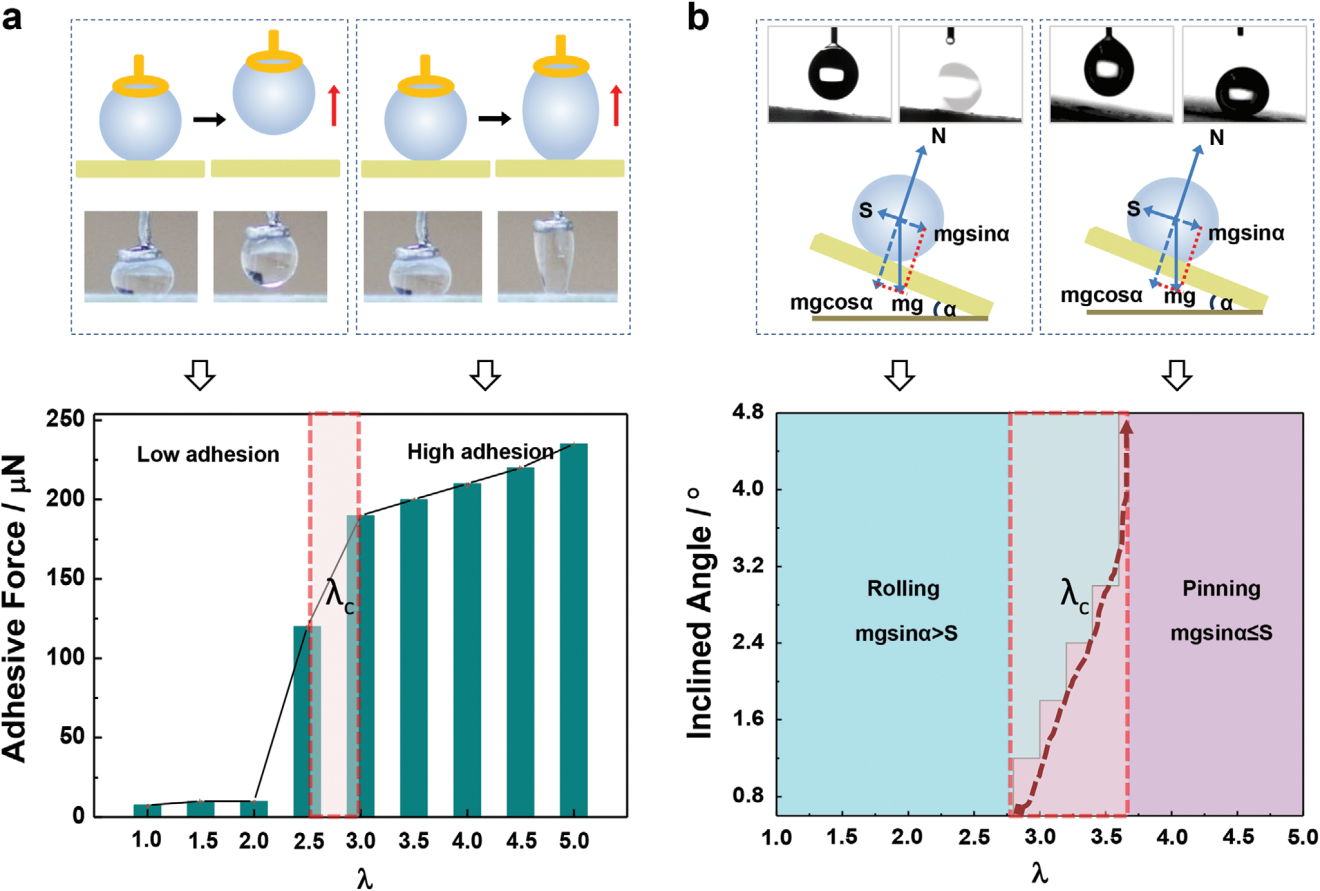
Dynamic wetting and adhesion behavior of the micro/nanostructured elastomer film. a) Water droplet (5 µL) adhesive performance on the micro/nanostructured elastomer film with varying equi‐biaxially stretch ratio. The surface adhesion increases with the increase of stretch ratio of the micro/nanostructured elastomer film, with a sudden rise at the critical stretch ratio range (*λ*_c_). b) The influence of tilt angle and stretch ratio of the micro/nanostructured elastomer film on the water droplet motion state. Water droplet can roll off the superhydrophobic elastomer film with slightly inclined angle and low stretch state owing to *mg *sin* α > S*, while droplet pins to the surface with high stretch state and larger distance of micro/nanostructured clusters due to *mg *sin* α ≤ S*. The results indicate that water droplet motion states can be controlled based on the significant change of water droplet adhesion on the micro/nanostructured elastomer film at *λ*_c_.

Besides, the micro/nanostructures of the elastomer film also have an important influence on the dynamic wetting and adhesion behavior of the surface. The adhesive force of a water droplet on the micro/nanostructured elastomer film with different equi‐biaxially stretch is measured. During the testing process, a droplet sticks on a copper ring initially, gradually contacts and is squeezed on the micro/nanostructured elastomer film. Then the droplet is lifted up and away from the micro/nanostructured elastomer film. With larger stretch ratio of the micro/nanostructured elastomer film, the droplet will stick onto the elastomer film surface and detach from the copper ring. These results indicate that with increased stretch ratio of the micro/nanostructured elastomer film, surface adhesion of the micro/nanostructured elastomer film to water increases. It is noticed that adhesive force has a sudden increase when the elastomer film is stretched from *λ* = 2.5 to *λ* = 3, which is the critical stretch ratio range (*λ*_c_) for the change from low to high (**Figure** **2**a).

Owing to the increase of the adhesive force with augmenting equi‐biaxially stretch of the micro/nanostructured elastomer film, the motion state of droplet on inclined film is varied (Figure 2b). The dynamic wetting behavior of droplet on the surface can be divided into two states, i.e., rolling and pinning. Water droplet can roll off the superhydrophobic elastomer film in slightly inclined angle and low stretch owing to *mg *sin* α > S* (where *m* is mass of water droplet, *g* is acceleration of gravity, *α* is tilt angle of micro/nanostructured elastomer film, and *S* is static friction force of micro/nanostructured elastomer film to droplet), while droplet pins to the surface with high stretch due to *mg *sin* α ≤ S* as the result of increased distance of micro/nanostructured clusters. These results indicate that the obvious change of wetting state for motion control on the elastomer surface is in the critical stretch ratio range (*λ*_c_) of *λ* = 2.8 to *λ* = 3.8, which provides the feasibility to dynamically control the droplet wetting states via electric field.

**Figure 3:**
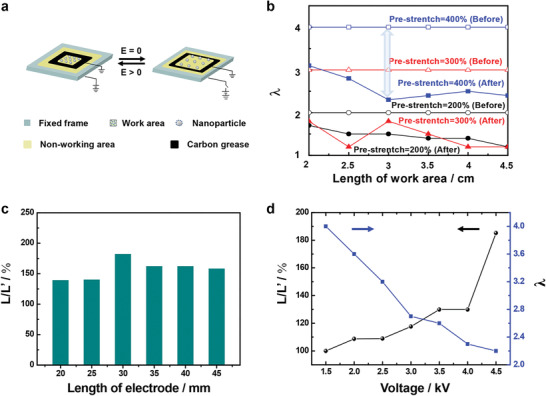
Electric field adaptive deformation of the micro/nanostructured elastomer film. a) Schematic of the electric field tuned micro/nanostructured elastomer film. The VHB film is firstly modified with the micro/nanostructured clusters of TiO_2_ and then fixed on the plastic frame after equi‐biaxially pre‐stretch. Then the carbon grease is evenly coated on both sides of elastomer film with a configuration like a Chinese character “
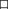
”, and the enclosed area serves as the work area for the following liquid droplet manipulation. When the voltage is applied on the electrodes, the elastomer film coated with carbon grease will expand and thus induce the contraction of the work area, leading to the shortening of the distance of micro/nanostructured clusters. b) Electric field induced stretch ratio change of the work area under different pre‐stretch ratios. When the pre‐stretch ratio of the work area changes from *λ*_p_ = 2 to *λ*_p_ = 4, the tunable stretch range of work area alters from *λ* = 1.3–2 to *λ* = 2.2–4 via electric field. c) The ratio of length of work area when *E* = 0 (*L*) over length of work area when *E* > 0 (*L*′) when *λ*_p_ = 4 is plotted as a function of different dimensions of work area. The length of work area decreases with the increase of applied voltage. When the work area is 30 × 30 mm, it shows the largest *L*/*L*′ of 180%. d) The ratio of *L*/*L*′ and stretch ratio *λ* changes with the different voltage under pre‐stretch of *λ*_p_ = 4. Taking the work area of 30 × 30 mm as an example, it shows the largest *L*/*L*′ of 180% under a 4.5 kV voltage, which is corresponding to the stretch ratio change of the work area from *λ* = 2.2 to *λ* = 4. The results indicate that the electric field can control the deformation of the elastomer film and the switch between Cassie state and Wenzel state of the droplet through applying different voltage is feasible.

Electric field induced stretching is the basic property of the dielectric elastomer. The dielectric elastomer film works like a flexible capacitor. When the electric field is applied on the flexible electrodes on both sides of the elastomer film, the elastomer turns into a flexible capacitor. Under the applied electric field across the electrodes, the electrostatic attraction between the opposite charges on opposing electrode and the repulsion of the same charges on each electrode generate stress on the film, causing it to reduce in thickness and expand in area. And the dielectric elastomer film can respond to external voltage almost simultaneously (a few milliseconds) and reach the maximum deformation within 40 ms (Figure S3, Supporting Information). In order to realize the electric field induced liquid motion on the micro/nanostructured elastomer film, the electric field controlled micro/nanostructured surface with superhydrophobicity is designed and shown in **Figure** **3**a. The very high bond (VHB) film is firstly modified with the micro/nanostructured clusters of TiO_2_ and then fixed on the plastic frame after equi‐biaxially pre‐stretch. Then the carbon grease is evenly coated on both sides of elastomer film with a configuration like a Chinese character “
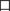
” and the enclosed area serves as the work area for the following liquid droplet manipulation (the unique design can effectively avoid the contamination and loss of the transported liquid), on which the deformation of the elastomer can be effectively enhanced by the cooperation of electric field and stretch. When the voltage is applied on the electrodes, the dielectric elastomer film coated with carbon grease will expand and thus induce the contraction of the work area, leading to the shortening of the distance of micro/nanostructured clusters.

To achieve the suitable transformation of the micro/nanostructured elastomer film via alternative electric field, the influence of the electrode area and voltage on the deformation of work area is investigated in detail in this work. When the equi‐biaxially pre‐stretch ratio *λ*_p_ varies from *λ*_p_ = 2 to *λ*_p_ = 4, the tunable stretch ratio range changes from *λ* = 1.3–2 to *λ* = 2.2–4 under the voltage range of 0–4.5 kV (Figure 3b). It is noting that the electric field induced stretch ratio of work area changes from *λ* = 2.2 to *λ* = 4 when elastomer film is in the pre‐stretch state of *λ*_p_ = 4, including the stretch state range of *λ* = 2.8 to *λ* = 3.8 for the droplet wettability and adhesion switch between low stretch state (*λ* < 2.8) and high stretch state (*λ* > 3.8) according to Figure 2b, and indicating that the motion state of droplet is expected to be controlled by electric field.

In addition, the dimensions of work area also has an important effect on the transformation through the electric field. Under the equi‐biaxially pre‐stretch of *λ*_p_ = 4, the ratio of *L* (expand length of work area when *E* = 0) over *L*' (contract length of work area when *E* > 0) with increasing applied voltage is studied (Figure 3c). The length of work area decreases with the increasing applied voltage. Taking the work area of 30 × 30 mm as an example, it shows the largest *L*/*L*′ of 180%, which is corresponding to the stretch change of the elastomer film from *λ* = 2.2 to *λ* = 4 (Figure 3d). The influence of electric field for the morphology of micro/nanostructured surface is studied. The results indicate that the electric field can tune the distance of micro/nanostructured clusters without changing the morphology of micro/nanostructured clusters (Figure S4, Supporting Information). Accordingly, the control of adhesion and dynamic wettability on the micro/nanostructured elastomer film is expected to be realized, and droplet motion state can be switched between rolling and pinning by adjusting the stretch ratio in a suitable range via applying different voltage, which provides the basis for droplet manipulation.

**Figure 4:**
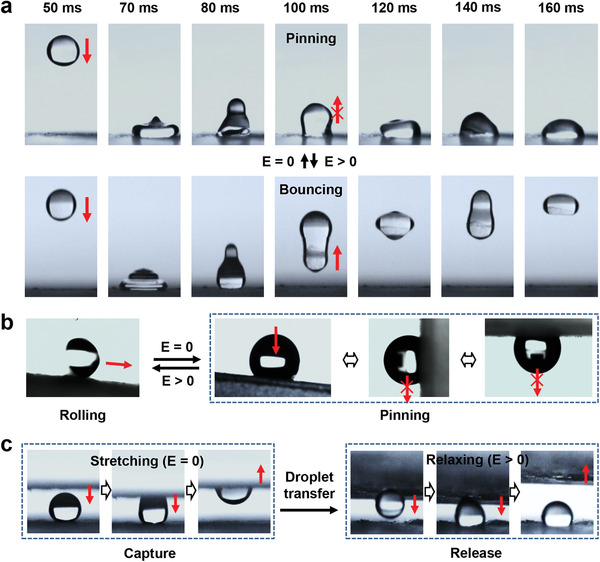
Demonstration of the dynamic liquid droplet manipulation and transportation on micro/nanostructured elastomer film via electric field. a) The control of water droplet bouncing behavior on the micro/nanostructured elastomer film surface via electric field. A droplet can bounce up to the height of 2 mm from the elastomer film with *λ*_p_ = 4 when *E* > 0, while the water droplet cannot bounce and will adhere to the surface as long as it approaches the surface when *E* = 0. b) Electric field controlled switch of the motion state of the droplet between rolling and pinning on the micro/nanostructured elastomer film. A droplet can roll down from the inclined micro/nanostructured elastomer film easily when the external electric field is applied (*E* > 0). Without the applied voltage, the droplet will pin on the film steadily, even with an inclined angle of 180°. c) Droplet transfer on the micro/nanostructured elastomer films with varying electric field. A droplet can be picked up without the external electric field, and then be precisely released under applied voltage after the droplet is moved to the desired area, which can be used as a tweezer to transfer a droplet. The results indicate that electric field induced deformation of elastomer film can be used to precisely control the liquid droplet dynamic wetting behavior and even the liquid transportation.

Similar to the dynamic wetting and adhesion behavior of the micro/nanostructured elastomer film controlled by mechanical stretch, the dynamic control of droplet motion states like jumping and pinning, catching and releasing, and controllable liquid transfer can be realized through the electric field in this work (**Figure** **4**). When a water droplet falls from the height of 15 mm and touches the elastomer film in equi‐biaxially pre‐stretch of *λ*_p_ = 4, the droplet can bounce up to the height of 2 mm and bounced several times owing to the contraction of the superhydrophobic micro/nanostructured elastomer surface when *E* > 0 (Figure S5, Supporting Information), while the water droplet cannot bounce and will adhere to the surface immediately as long as it approaches the surface due to the large distance of clusters when *E* = 0 (Figure 4a). The results indicate that the switch of droplet wetting behavior from bouncing to catching can be controlled by electric field owing to the wetting state fast transition from Cassie state to Wenzel state. Meantime, liquid droplet motion state can be switched between rolling and pinning by electric field when a droplet is put onto the micro/nanostructured elastomer film. A water droplet can roll down from the inclined elastomer film easily when external electric field is applied, while the droplet will pin on elastomer film steadily without the applied voltage even when the surface is inverted (Figure 4b). Therefore, the surface can capture the droplet under high adhesion state and release the droplet as the result of low adhesion, which can be used as a “droplet tweezer” for the lossless and contamination‐free droplet transfer. As shown in Figure 4c, the micro/nanostructured elastomer film can lift a droplet from a superhydrophobic surface due to the high surface adhesion to overcome the droplet gravity when *E* = 0, and then release the droplet due to the reduced adhesion force when *E* > 0. Nearly lossless transfer of water droplet can be achieved using the stretchable micro/nanostructured elastomer film. Therefore, the electric field‐induced stretchable elastomer film can be used for lossless water droplet transportation, which can serve as a useful tool for surface microfluidics and may have many potential applications that are related to droplet handling and transfer.

## 3 Conclusion

In conclusion, a strategy for achieving controllable liquid manipulation and transportation on a micro/nanostructured elastomer film via electric field is proposed. Based on the electrostriction of the dielectric elastomer film, the surface wetting state can be transformed from Cassie state to Wenzel state and adhesion can be adjusted fastly via transforming the spacing of the micro/nanostructured clusters on the elastomer film. Correspondingly, the motion states of the droplets on the superhydrophobic elastomer surface can be controlled dynamically, such as jumping and pinning, catching and releasing, and controllable liquid transfer without loss and contamination, via adjusting electric field. Thus this work stands for a different concept from previous studies of the fixed traditional superhydrophobic surfaces, and provides a new route to achieve dynamic liquid droplet manipulation and transportation. The ability of the micro/nanostructured elastomer film may provide opportunities for designing and constructing new class of materials for various applications. For example, in the foggy environment, it can collect water when the voltage is off, but can play the role of anti‐fog and anti‐ice when voltage is on. A micro‐reaction device can be designed to transfer the solution droplet and mix the reactants. Such mechanism can also be used to make responsive pipe to control the transported liquid according to different requirements.

## 4 Experimental Section

##### Fabrication of the Micro/Nanostructured Elastomer Film

TiO_2_ nanoparticles were modified to be hydrophobic by steaming process. A clean and dry culture dish (about 160 mm in diameter) was used to distribute 0.4 mg TiO_2_ nanoparticles evenly on the surface, and a small piece of glass (20 × 20 mm) was placed in the center of dish, and 2–3 drops of perfluorodecyl trimethyl siloxane were dripped on the glass sheet through a pipette, sealed and placed on the heating plate at 100 °C for 8 h. The hydrophobic TiO_2_ nanoparticles were adhered to the VHB 4910 film (3M Company, VHB stands for very high bond. 3M VHB 4910 is a kind of foam‐based material with double‐sided adhesive and the thickness of ≈1 mm, which belongs to VHB adhesive system (pressure‐sensitive adhesive system).) to form superhydrophobic surface with the density of 0.01 mg cm^−2^.

##### Construction of the Electric Field Tuned Micro/Nanostructured Elastomer Device

The hydrophobic TiO_2_ nanoparticles were firstly adhered to the VHB 4910 film (3M Company). The film was then equi‐biaxially pre‐stretched and fixed to a plastic frame. The carbon grease was coated onto the two sides of the pre‐stretched VHB film as electrodes in a configuration like a Chinese character “
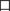
”. The copper tapes were contacted with the carbon grease and connected to the high voltage power supply.

##### Instrument Characterization

Scanning electron microscope (SEM) images were obtained using a field‐emission SEM at 10 kV (Quanta 250 FEG). Water (5 µL) CAs were measured using a CA measurement instrument (OCA 20, Data‐Physics) at ambient temperature. Each reported CA was an average of at least five independent measurements. The water droplet adhesive forces were measured by a high‐sensitivity microelectromechanical balance system (Data‐Physics DCAT 11, Germany). A 5 µL water droplet was suspended with a cooper loop and controlled to contact with the elastomer film to the micro/nanostructured elastomer film at a constant speed of 0.005 mm s^−1^ and then to leave at a speed of 0.005 mm s^−1^. Analysis of the droplet bounce and transfer on the micro/nanostructured elastomer film was recorded with the high speed camera (Revealer 2F01M) from the side view.

## Conflict of Interest

The authors declare no conflict of interest.

## Supporting information

Supporting Information
