# Supplementary material for: Switchable Wettability and Adhesion of Micro/Nanostructured Elastomer Surface via Electric Field for Dynamic Liquid Droplet Manipulation
Source: Adv Sci (Weinh). 2020 Aug 2;7(18):2000772. doi: 10.1002/advs.202000772 (PMC7509640; doi:10.1002/advs.202000772)
Supplement: Supplementary file 1 — Supporting Information [file ADVS-7-2000772-s001.pdf]

© 2020 Wiley-VCH GmbH

ADVANCED SCIENCE
Open Access

Supporting Information

for *Adv. Sci.*, DOI: 10.1002/advs.202000772

Switchable Wettability and Adhesion of Micro/Nanostructured Elastomer Surface via Electric Field for Dynamic Liquid Droplet Manipulation

Yan Li, Jinrong Li, Liwu Liu, Yufeng Yan, Qiuya Zhang, Na Zhang, Linlin He, Yanju Liu, Xiaofang Zhang, Dongliang Tian, Jinsong Leng,* and Lei Jiang*

Supporting Information

Switchable Wettability and Adhesion of Micro/Nanostructured Elastomer Surface via Electric Field for Dynamic Liquid Droplet Manipulation

Yan Li, Jinrong Li, Liwu Liu, Yufeng Yan, Qiuya Zhang, Na Zhang, Linlin He, Yanju Liu, Xiaofang Zhang, Dongliang Tian, Jinsong Leng,* and Lei Jiang*

Dr. Y. Li, Y. Yan, Dr. Q. Zhang, N. Zhang, L. He, Prof. D. Tian, Prof. L. Jiang
Key Laboratory of Bio-Inspired Smart Interfacial Science and Technology of Ministry of Education
School of Chemistry, Beihang University, Beijing 100191, P. R. China
E-mail: tiandl@buaa.edu.cn
Prof. D. Tian, Prof. L. Jiang
Beijing Advanced Innovation Center for Biomedical Engineering
Beihang University, Beijing 100191, P. R. China
Dr. J. Li, Prof. J. Leng
National Key Laboratory of Science and Technology on Advanced Composites in Special Environments
Harbin Institute of Technology, Harbin, Heilongjiang 150080, P.R. China
E-mail: lengjs@hit.edu.cn
Prof. L. Liu, Prof. Y. Liu
Department of Astronautical Science and Mechanics
Harbin Institute of Technology, Harbin, Heilongjiang 150001, P.R. China
Prof. X. Zhang
School of Mathematics and Physics
University of Science and Technology Beijing, Beijing 100083, P. R. China
Prof. L. Jiang
Technical Institute of Physics and Chemistry
Chinese Academy of Sciences, Beijing 100191, P. R. China

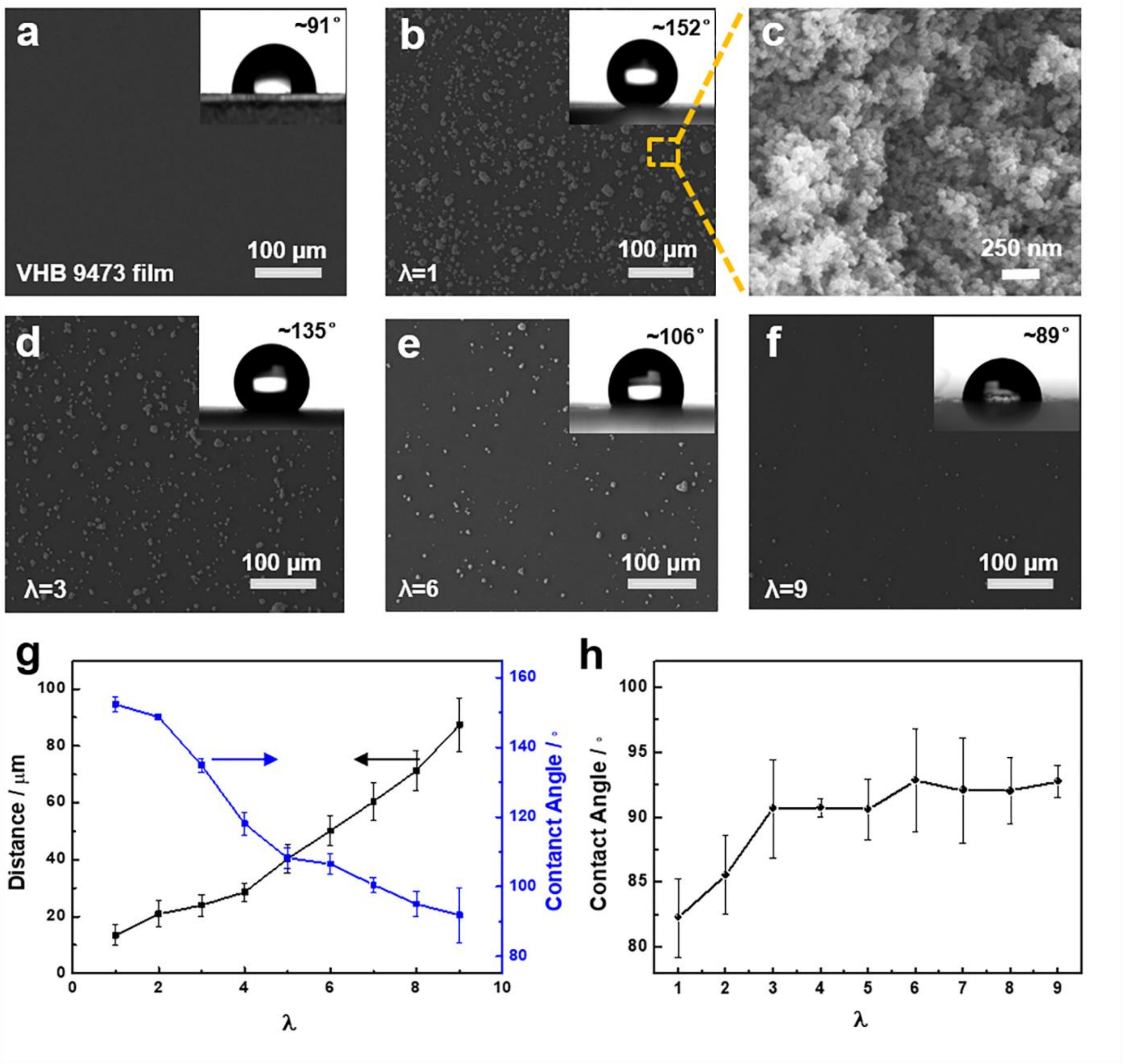

Figure S1 Morphology and wettability of the micro/nanostructured elastomer film under different equi-biaxially stretch ratios. (a) SEM image of the original elastomer film (VHB 4910). (b-c) SEM images of micro/nanostructured clusters on the elastomer film under non-stretch state. (d-f) The morphology of micro/nanostructured elastomer film under different stretch ratios (d) λ=3, (e) λ=6, (f) λ=9. The insets are water contact angle (CA) photographs of the corresponding micro/nanostructured elastomer film. (g) Distance of micro/nanostructured clusters and water CA change with stretch ratio λ. With the increasing of stretch ratio, the distance of micro/nanostructured clusters increases, while the water CA decreases. (h) Water CAs of the original elastomer film (VHB 9473) with different stretch ratio λ. The water CAs increase from ~83° to ~90° with the change of stretch increasing from λ=1 to λ=9, and the water CAs gradually reach a plateau after the stretch λ>3.

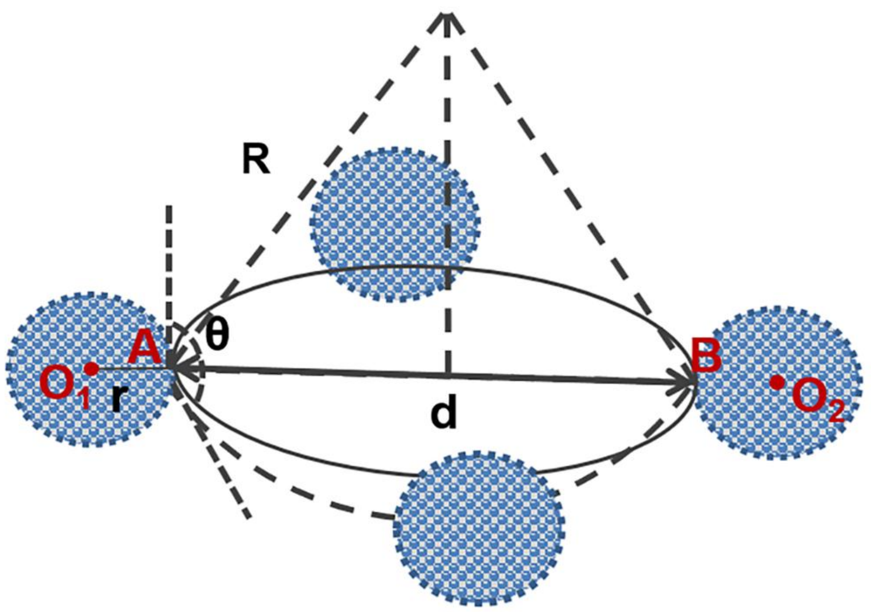

Figure S2 Schematic diagram of water droplet wetting on the micro/nanostructured elastomer film. For the micro/nanostructured elastomer film with the radius of micro/nanostructured clusters (r), water CA (θ), radius of water (R) and the distance of micro/nanostructured clusters (d), based on the relationship

$$\mathrm{R} = \frac{r}{1 - \sin(\pi - \theta)} = \frac{r}{1 - \sin\theta}$$

$$\frac{d/2}{R} = \cos(\pi - \theta) = -\cos(\theta)$$

The critical distance of micro/nanostructured (d_c) clusters when the droplet just contacts with elastomer film can be calculated as

$$d_c = \frac{2r\cos\theta}{\sin\theta - 1}$$

For the micro/nanostructured elastomer film with water CA of ~152°, and r=6.7 ± 1.5 μm, the distance of micro/nanostructured cluster (AB) by calculation is 22.3 ± 5.0 μm, i.e. the center distance of micro/nanostructured cluster (O_1O_2) is 35.7 ± 5.0 μm, the droplet will contact with the substrate and just wet elastomer film.

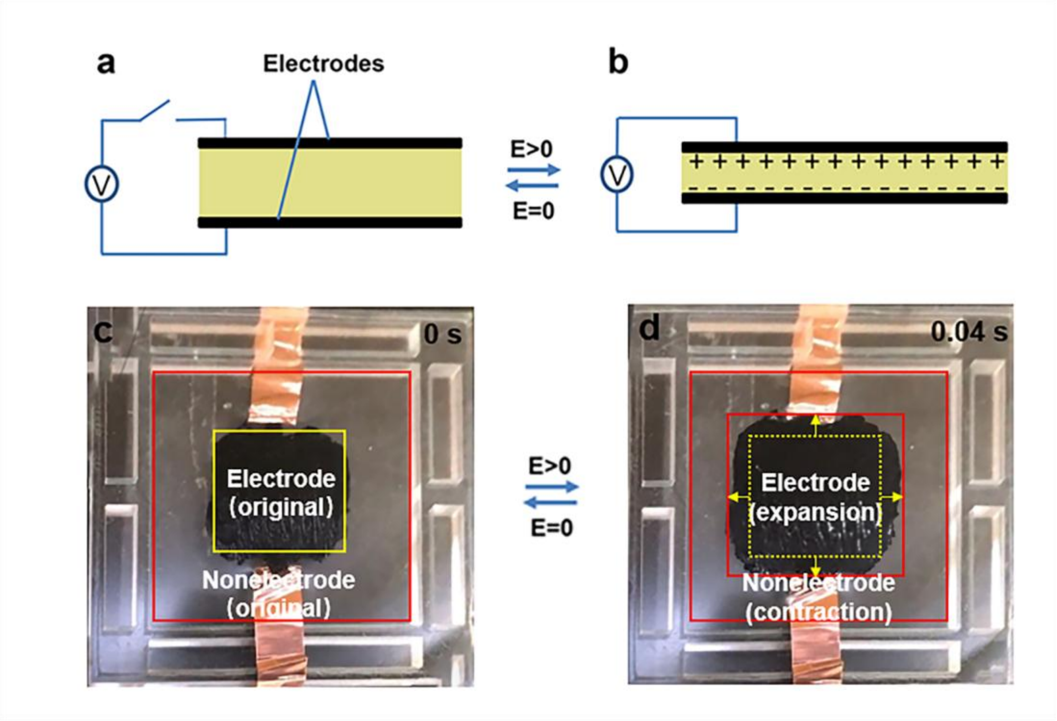

Figure S3 Electric field induced rapid deformation of the dielectric elastomer film. (a-b) Deformation principle of the dielectric elastomer film. The dielectric elastomer film works like a flexible capacitor. When the electric field is applied on the flexible electrodes on both sides of the elastomer film, the elastomer turns into a flexible capacitor. Under the applied electric field across the electrodes, the electrostatic attraction between the opposite charges on opposing electrode and the repulsion of the same charges on each electrode generate stress on the film, causing it to reduce in thickness and expand in area. (c-d) The dielectric elastomer film can respond to external voltage almost simultaneously and reach the maximum deformation (electrode area expansion and nonelectrode area contraction) in 40 ms.

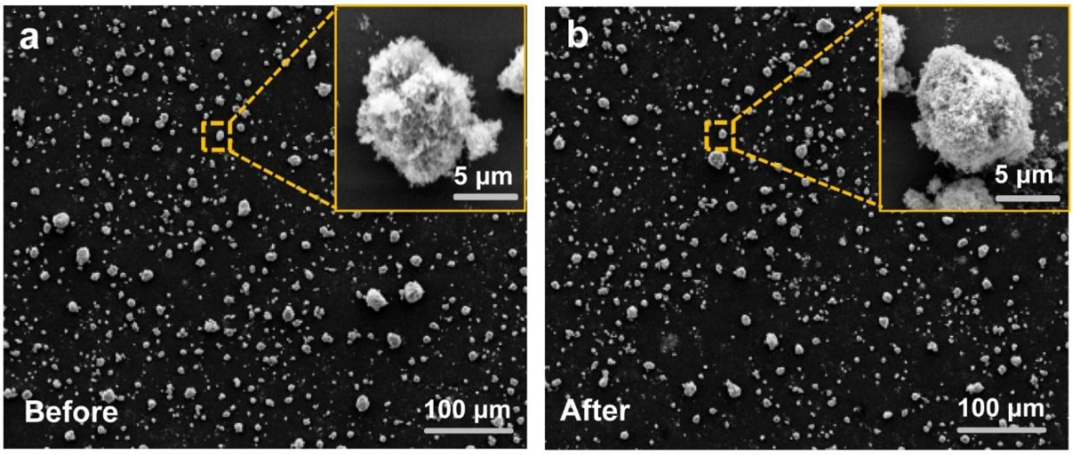

Figure S4 SEM images of the micro/nanostructured elastomer film before applied voltage (a) and after power dump (b). The results indicate that the electric field has no effect on morphology of micro/nanostructured clusters.

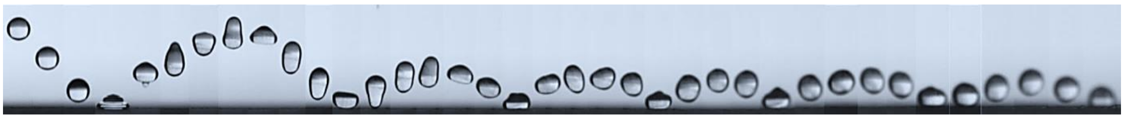

Figure S5 The continuous bouncing behavior of water droplet. A droplet can bounce up several times from the elastomer film when E>0.
